# Prevalence of Cognitive Impairment in Recently Diagnosed Type 2 Diabetes Patients: Are Chronic Inflammatory Diseases Responsible for Cognitive Decline?

**DOI:** 10.1371/journal.pone.0141325

**Published:** 2015-10-30

**Authors:** Pilar Lavielle, Juan O. Talavera, Nancy Reynoso, Marissa González, Rita A. Gómez-Díaz, Miguel Cruz, Felipe Vázquez, Niels H. Wacher, DIMSS Study Group

**Affiliations:** 1 Unidad de Investigación en Epidemiología Clínica UMAE Hospital de Especialidades, Centro Médico Nacional Siglo XXI, Instituto Mexicano del Seguro Social (IMSS), Mexico City, México; 2 Unidad de Investigación en Bioquímica, UMAE Hospital de Especialidades, Centro Médico Nacional Siglo XXI, Instituto Mexicano del Seguro Social (IMSS), Mexico City, México; Banner Alzheimer's Institute, UNITED STATES

## Abstract

**Objective:**

To estimate the prevalence of cognitive impairment (CI) among patients recently diagnosed with type 2 diabetes (RDD) and to identify any relationships between CI and RDD comorbidities. Methods: One thousand seven hundred twelve patients with RDD participated in a cross-sectional study. The patients’ sociodemographic and clinical data were registered.

**Results:**

The sample population had an average age of 51 ± 11 years, and 63.26% of the patients were female. CI was diagnosed in 38 patients (2.2%) and was more common among both females (2.8% vs. 1.3%, *p* = 0.063) and the elderly (0% at an age ≤ 30 years vs. 10.4% at an age > 70 years, *p* = 0.0001). Rheumatoid arthritis (present in 15.8% vs. absent in 2.1%) and asthma (13% vs. 2.1%) correlated significantly with CI based on the results of our logistic regression analysis.

**Conclusion:**

Age, female gender, rheumatoid arthritis and asthma are risk factors for CI in the setting of RDD.

## Introduction

A decline in cognitive ability results in the loss of independent function and may progress to dementia. For this reason, decreased cognitive function has a considerable impact at the individual, familial and institutional levels [1]. Progress has been made during the past decade in recognizing the factors that may increase the risk of developing cognitive impairment (CI) [1], including “classic” cardiovascular factors (e.g., hypertension and hyperlipidemia), which have been used for the creation of indices pertaining to the risk of dementia. These instruments combine information about known risk factors applied to statistical models using a weighted point system to predict an individual’s risk of developing a particular disease, which may be used in both clinical medicine and research [2,3]. However, additional factors have been difficult to identify, as it has not been determined whether these factors are indeed causal factors with respect to cognitive deterioration such as gender, lifestyle (physical and mental activity), smoking, alcohol consumption, psychosocial factors (social activity), vitamin D deficiency, testosterone deficiency, and subclinical thyroid dysfunction [4,5].

During the earliest stages of CI, patients exhibit deficits in both attention and concentration; difficulty with memory, executive function and basic calculations; and a reduction in the speed at which they process information [6,7].

The prevalence of CI without dementia has been estimated as 22.2% among individuals ≥ 70 years of age in the United States and as 38.6% among patients from 90–95 years of age. Furthermore, previous studies have suggested that CI may progress to full dementia at a rate of 10% to 15% per year [8,9].

Type 2 diabetes (T2D) appears to be a risk factor for CI. Despite differences in study designs and problems with study methodologies, most studies have demonstrated that individuals with T2D exhibit poor cognitive function and that the prevalence of dementia is twice that observed among individuals without diabetes [10].

In patients with diabetes, the pathogenesis of CI is most likely multifactorial. The alterations that occur in patients with CI have been linked to macrovascular changes; demographic variables, such as age and female gender; metabolic syndrome; and recurrent bouts of severe hypoglycemia [11–15].

Physicians in primary care seldom screen the cognitive abilities of patients [16]. It is possible that the same occurs among patients with diabetes, making it likely that CI is under-diagnosed among patients with diabetes. Therefore, interventions are not often implemented to improve the cognitive function of patients with type 2 diabetes.

However, because of the increasing prevalence of type 2 diabetes, understanding both the frequency and the possible causes of diabetes-related cognitive impairment is necessary [17]. This importance stems from the fact that CI reduces quality of life, and may cause neuropsychiatric symptoms and disabilities to worsen, increasing health care costs [17].

The purpose of this study was to estimate the prevalence of CI among patients recently diagnosed with type 2 diabetes and to determine whether diabetes-related co-morbidities are associated with a higher prevalence of CI [18, 19].

## Materials and Methods

### Setting

After receiving approval from the CMN Siglo XXI ethics committee for both the study and the informed consent letter (IMSS-2004-131), a cross-sectional analytical survey was performed.

This study was performed during the initial clinical evaluation of a cohort of patients with diabetes to evaluate various clinical and genetic aspects of the disease. A sufficiently sized sample was selected from the largest and most well-organized healthcare system in Mexico (the Instituto Mexicano del Seguro Social (IMSS)). IMSS coverage includes all formally employed individuals and their families, a population of approximately 50 million people (nearly one half of the population). For this study, we included only primary care clinics in Mexico City, where diabetes patients are customarily diagnosed and treated.

### Sample

The sample was selected from a census of patients with diabetes from the 10 family medicine clinics selected for the study. Patients were scheduled for visits to the clinic via phone calls. Patients were included if they met the following inclusion criteria: older than 18 years of age, a Karnofsky functional status ≥ 80, and recently diagnosed (< 3 years) with type 2 diabetes according to the American Diabetes Association criteria (plasma glucose ≥ 126 mg/dL on at least 2 occasions) [20]. Patients with psychiatric conditions, depression, anxiety, alcohol or substance abuse, psychosis, active neoplasms, HIV/AIDS, liver cirrhosis, previous stroke, or previous glucocorticoid use were excluded. Following an explanation of the study, all participants signed an informed consent letter.

Of the 3596 candidates in the census, 1647 were not included in the study, either because they declined to participate (n = 184) or because the original sample size had been met.

### Procedure

During the initial stages of the study, every patient was subjected to a complete evaluation (medical history, physical examination and chart review). The baseline questionnaire included every previous diagnosis for each patient as determined by a primary care physician (such as hypertension, cardiovascular disease, bone fractures, cancer, and psychiatric disease), as well as any drug treatments, diabetes complications and cognitive tests. The evaluations were performed by a multidisciplinary team in the Clinical Epidemiology Research Unit at the National Medical Center.

### Study variables

#### I Outcome variable

Cognitive impairment was evaluated based on a screening assessment consisting of 3 tests, each of which was associated with a 1-point value. We estimated that failure of at least 2 of the tests corresponded to moderate CI. The psychometric properties of 3 tests were grouped into a single construct of “moderate cognitive impairment.” Factor analysis was utilized to determine construct validity. The 3 tests were grouped into a single dimension that explained 52% of the variance, with good correlations with the interior of the factor space (from 0.623 to 0.793).

I.1: The clock drawing test free-hand format (CDT) is a simple and effective means of performing a neuropsychiatric assessment of patients. The research literature supports its use as a reliable screening tool for cognitive dysfunction, particularly dementia [21–23]. The clock-drawing test evaluates visuospatial and executive function, attention, comprehension and numeric knowledge. To determine the presence of CI, we considered patients normal if they scored ≥6.

I.2: Verbal fluency. This is a subtest of the Qmcil (Quick Mild Cognitive Impairment) and is commonly used to distinguish between intermediate CI and normal cognition. The verbal fluency test is scored based on the words that the patient evokes and the time necessary for the patient to do so. For our test, the patient was asked to name each animal that either he or she remembers, as fast as possible, with a median score of 20 in one minute. The patients were considered normal if they were able to name ≥ 7 animals [24–26].

I.3: Calculation (successive subtractions). This test is useful for evaluating the cognitive abilities of individuals of lower socioeconomic status. The patient was asked to successively subtract the same number from a beginning number, with a maximum possible score of 12. For our study, the patients were considered normal if they performed ≥ 6 consecutive subtractions [27, 28]. These tests were previously compared using the WAIS (Wechsler Adult Intelligence Scale) in 40 patients (28 women) with an average age of 52 years (range of 24 to 80) and an average educational level of 5.78 years (range of 0 to 16 years). Using Kappa testing, a level of agreement of 0.63, a sensitivity of 45% and a specificity of 83% were observed for the calculation test [29].

#### II. The exposure variables

The following variables were analyzed with respect to their relationship with CI: age, gender, fasting glucose levels, diabetes-associated co-morbidities (e.g., hypertension, dyslipidemia and ischemic cardiovascular disease) and co-morbidities not associated with diabetes (e.g., a history of neoplasms, asthma, COPD and rheumatic disease, including rheumatoid and degenerative arthritis).

### Data analysis

Continuous variables are presented as the means ± standard deviations. The relationship between each condition and CI was assessed using the chi-square test. Furthermore, all variables were included in a backward stepwise multiple logistic regression model in which a *p*-value of < 0.10 was considered for inclusion.

## Results

For our analysis, we considered 1,943 patients, 231 of whom were excluded due to depression, anxiety, previous cerebrovascular disease, alcohol or substance abuse, psychosis, active neoplasms, HIV/AIDS, liver cirrhosis, glucocorticoid use and limitations in their daily activities due to either pain or the sequelae of an underlying disease.

The final study sample consisted of 1,712 patients recently diagnosed (< 3 years) with type 2 diabetes. Of these patients, 1,083 (63.26%) were female. The mean age of the cohort was 51 ± 11 years, and the average level of education was 6 years (5.4% were college graduates).

CI was diagnosed in 38 (2.2%) patients. CI was more common among older individuals (0% at an age ≤ 30 years versus 10.4% at an age > 70 years).

No differences were observed in the prevalence of CI when the sample was stratified according to hypertension, dyslipidemia, ischemic heart disease or glycemic control. Table 1 also includes the relationships between CI and conditions not related to metabolic syndrome. CI was significantly more common among patients with rheumatoid arthritis (absent 2.1% vs. present 15.8%) and asthma (absent 2.1% vs. present 13%).

**Table 1:** Demographic characteristics and conditions of patients with type 2 diabetes and their relationship with cognitive impairment.

Variable	Cognitive impairment (%)	Odds ratio (IC 95%)	P value
Gender			
Male	8/629 (1.3)	2.21	0.063
Female	30/1083 (2.8)	(0.96–5.26)	
Age (years)			
≤ 30	0/52 (0.0)	8.99	0.0001
31–50	10/781 (1.7)	(2.96–26.81)	
51–70	21/812 (2.4)		
≥ 70	7/67 (10.4)		
Education			
≤ 6	24/1040 (2.3)	1.11	0.79
≥ 7	14/672 (2.0)	(0.55–2.28)	
Glucose levels (mmol/L)			
≤ 6.9	1/43 (2.3)	0.95	0.96
≥ 7.0	37/1669 (2.2)	(0.14–19.10	
Hypertension			
No	24/1170 (2.1)	1.27	0.48
Yes	14/542 (2.6)	(0.62–2.57)	
Dyslipidemia			
No	30/1407 (2.1)	1.24	0.75
Yes	8/305 (2.6)	(0.52–2.85)	
Ischemic heart disease			
No	37/1685 (2.2)	1.71	0.45
Yes	1/27 (3.7)	(0.04–11.02)	
History of neoplasm			
No	38/1700 (2.2)	---------	0.76
Yes	0/12 (0.0)		
Chronic bronchitis			
No	37/1685 (2.2)	1.71	0.45
Yes	1/27 (3.7)	(0.04–11.0)	
Asthma			
No	35/1689 (2.1)	7.09	0.013
Yes	3/23 (13.0)	(1.06–26.83)	
Rheumatoid arthritis			
No	35/1693 (2.1)	8.88	0.008
Yes	3/19 (15.8)	(1.96–34.50)	
Degenerative arthritis			
No	32/1558 (2.1)	1.93	0.13
Yes	6/154 (3.9)	(0.71–4.94)	

We subsequently conducted a backward stepwise multiple logistic regression analysis in which all of the variables that were significant in the bivariate analysis remained significant in the multivariate model. These variables included age (OR 1.05 [1.02–1.08]), rheumatoid arthritis (OR 5.16 [1.32–20.35]) and asthma (OR 5.11 [1.32–19.73]).

## Discussion

This study was conducted to determine the prevalence of CI among patients recently diagnosed with T2D and to investigate the possible relationships between CI with both T2D and non-T2D comorbidities.

The prevalence of CI among patients recently diagnosed with type 2 diabetes in our study was 2.2%, which is lower than that reported in previous studies. A possible explanation for this difference may be the ages of the patients included in the analyses, as the patients in our study were relatively young. For example, the estimated prevalence of CI among patients ≥ 65 years old is 7% in Mexico and may vary between 3% and 5.9% in Latin America [30]; and the relatively low prevalence of CI noted among the patients with T2D in our study is consistent with the results of studies analyzing younger patient cohorts [31]. However, the prevalence noted in the present study was higher than expected for a group of patients with an average age of 50 years.

In our analysis, CI did not appear to be related to any of the signs and symptoms of metabolic syndrome. These results differ from most studies that have observed a relationship between hypoglycemia and CI, whereas other studies have detected a relationship between the components of metabolic syndrome and CI [32].

Our results suggest that CI is associated with conditions that are apparently unrelated to metabolic syndrome, such as rheumatoid arthritis and asthma. These relationships may be explained by the fact that both diseases are characterized by chronic inflammatory processes [33,34]. Moreover, different studies have noted that low-grade inflammation may compromise the integrity of cerebral function [35–42].

Studies have shown that obesity, pre-diabetes and diabetes are associated with low-grade inflammation. Furthermore, the early stages of diabetes are often asymptomatic, and the disease may be diagnosed long after its onset (several years). However, obesity may precede diabetes by several years. Despite the cross-sectional design of our study, the relationships observed between the evaluated risk factors and CI support the hypothesis that inflammatory conditions play a role in the development of CI among patients with type 2 diabetes.

A limitation of our study involved the use of screening tests to detect cognitive impairment. However, by using a cut-off point of at least 2 abnormal test scores, we ensured a high degree of specificity, even if less severe CI was not detected via this method.

Other common risk factors for CI (e.g., hypoglycemia and cardiovascular disease) may not have been identified because (i) the patients were recently diagnosed with T2D and (ii) most of the patients exhibited a moderate to high degree of hyperglycemia. The traditional risk factors may become evident only following a longer period of time.

Despite the relatively large sample size of our study, only a limited number of patients suffered from either asthma or arthritis, and the 95% confidence intervals for the odds ratios associated with these co-morbidities were large. However, the odds ratios were sufficiently large to indicate a statistically significant relationship.

In conclusion, our results indicated (i) that the prevalence of CI in Mexican patients recently (≤3 years) diagnosed with diabetes was 2.2% and (ii) that chronic inflammatory diseases such as asthma and arthritis are risk factors for CI among these patients.

Therefore, additional studies investigating strategies (such as regular exercise) that may protect against the deleterious effects of inflammation in these patients are necessary.

## References

[1] Langa K, Larson E, Karlawish, Cutler D, Kabeto M, Kim S, et al Trends in the prevalence and mortality of cognitive impairment in the United States: Is there evidence of a compression of cognitive morbidity? Alzheimer’s & Dementia 2008; 4: 134–144. 10.1016/j.jalz.2008.01.001 PMC2390845 18631957

[2] Barnes D, Covinsky K, Whitmerd R, Kullere L, Lopez O, Yaffe K. Dementia Risk Indices: A Framework for Identifying Individuals with a High Dementia Risk. Alzheimers Dement. 2010; 6: 138–141141. 10.1016/j.jalz.2010.01.005 20298975 PMC2909695

[3] Llewellyn D, Lang I, Xie J, Huppert F, Melzer D, Langa L. Framingham Stroke Risk Profile and poor cognitive function: a population-based study. BMC Neurology 2008, 8:12 10.1186/1471-2377-8-12 Available: http://www.biomedcentral.com/1471-2377/8/12) 18430227 PMC2386808

[4] Barnes D, Yaffe K. Predicting dementia: Role of dementia risk índices. Future Neurol. 2009;4(5): 555–560. 10.2217/fnl.09.43 20161571 PMC2805956

[5] Etgen T, Sander D, Bickel H, Förstl H. Mild cognitive impairment and dementia: the importance of modifiable risk factors. Dtsch Arztebl Int 2011; 108(44): 743–50. 10.3238/arztebl.2011.0743 22163250 PMC3226957

[6] American Psychiatric Association DSM-IV. Diagnostic and Statistical Manual of Mental Disorders. Fourth Edition Washington D. C. 1993.

[7] Reijmer Y, Brundel M, De Bresser J, Kappelle J, Leemans A, Biessels G. On behalf of the Utrecht vascular cognitive impairment study group. Microstructural white matter abnormalities and cognitive functioning in Type 2 Diabetes. Diabetes Care 2013; 36:137–144 10.2337/dc12-0493 22961577 PMC3526236

[8] Melzer D, Ely M, Brayne C. Cognitive impairment in elderly people: Population based estimate of the future in New England, Scotland and Wales. BMJ 1997; 315:462 9284665 10.1136/bmj.315.7106.462 PMC2127304

[9] Plassman B, Langa K, Fisher G, Heeringa S, Weir D, Ofstedal M, et al Prevalence of Cognitive Impairment without Dementia in the United States. Ann Intern Med. 2008, 18; 148(6): 427–434. 18347351 10.7326/0003-4819-148-6-200803180-00005 PMC2670458

[10] Luchsinger J. Diabetes, Related Conditions, and Dementia. J Neurol Sci. 2010 12 15; 299(1–2): 35–38. 10.1016/j.jns.2010.08.063 20888602 PMC2993820

[11] Di Carlo A, Baldereschi M, Amaducci M. Cognitive impairment without dementia in older people: Prevalence, vascular risks factors, impact on disability. The Italian longitudinal study on aging. J Am Ger Soc 2000; 48:775–782. 10.1111/j.1532-5415.2000.tb04752.x 10894316

[12] Hoffman R, Speelman D, Hinnen D. Changes in cortical functioning with acute hypoglycemia and hyperglycemia in type I diabetes. Diabetes Care 1989; 3:193–197. 10.2337/diacare.12.3.193 2702910

[13] Langan S, Deary I, Jepburn D. Cumulative cognitive impairment following recurrent severe hypoglycemia in adult patients with insulin treated diabetes mellitus. Diabetologia 1991; 34:337–344. 1864488 10.1007/BF00405006

[14] Ebady S, Arami M, Shafigh M. Investigation on the relationship between diabetes mellitus type 2 and cognitive impairment. Diabetes Res Clin Pract 2008; 82:305–309. 10.1016/j.diabres.2008.08.020 18848366

[15] Brands A, Biessels G, De Haan E, Kappelle J, Kassel R. The Effects of Type 1 Diabetes on Cognitive Performance: A meta-analysis. Diabetes Care 2005 28(3): 726–735. 15735218 10.2337/diacare.28.3.726

[16] Boustani M, Peterson B, Hanson L, Harris R, Lohr K. Screening for dementia in primary care: A Summary of the evidence for the U.S. Preventive Services Task Force. Ann Intern Med 2003;138:927–937. 12779304 10.7326/0003-4819-138-11-200306030-00015

[17] Strachan M, Frier B, Deary I. Type 2 diabetes and cognitive impairment. Diabetic Medicine 2003; 20:1–2. 10.1046/j.1464-5491.2003.00855.x 12519313

[18] Roberts R, Geda Y, Knopman D, Christianson T, Pankratz V, Boeve B, et al Association of duration and severity of diabetes mellitus with mild cognitive impairment. Arch Neurol 2008; 65:1066–1076. 10.1001/archneur.65.8.1066 18695056 PMC2630223

[19] Whitmer R. Type 2 diabetes and risk of cognitive impairment and dementia. Curr Neurol Neurosci Rep 2007; 7:373–380. 17764626 10.1007/s11910-007-0058-7

[20] American Diabetes Association Standards of Medical Care in Diabetes—2009. Diabetes Care 2009; 32 (SUPPL 1): S13–S61. 10.2337/dc09-S013 19118286 PMC2613589

[21] Wolf-Klein G, Silverstone F, Levy A. Screening for Alzheimer´s disease by Clock Drawing. J Am Geriatr Soc 1989; 37:730–734. 2754158 10.1111/j.1532-5415.1989.tb02234.x

[22] Brodaty H, Moore C. Clock drawing test for dementia of the Alzheimer type: a comparison of three scoring methods in a memory disorders. Clinic International Journal of Geriatric Psychiatry 1997; 12:619–627. 9215942

[23] Eknoyan D, Hurley R, Taber K.The Clock Drawing Task: Common errors and functional neuroanatomy. J Neuropsychiatry Clin Neurosci 2012; 24:3 Available: http://neuro.psychiatryonline.org 10.1176/appi.neuropsych.12070180 23037640

[24] O’Caoimh R, Gao Y, McGlade C, Healy L, Gallagher P, Timmons S, Molloy D. Comparison of the quick mild cognitive impairment (Qmci) screen and the SMMSE in screening for mild cognitive impairment. Age and Ageing 2012; 41: 624–629. 10.1093/ageing/afs059 22610464 PMC3424052

[25] O'Caoimh R, Gao Y, Gallagher P, Eustace J, McGlade C, Molloy DW. Which part of the Quick Mild Cognitive Impairment Screen (Qmci) discriminates between normal cognition, mild cognitive impairment and dementia? Age & Ageing 2013; 42(3):324–30. 23612864 10.1093/ageing/aft044 PMC3633367

[26] Juncos O, Facal D, Lojo C, Pereiro A. Does tip-of-the-tongue for proper names discriminate amnestic mild cognitive impairment? International Psychogeriatrics 2013; 25(4):627–34. 10.1017/S1041610212002207 23253431

[27] Apolinario D, Brucki S, Ferretti R, Farfel J, Magaldi R, Busse A, et al Estimating premorbid cognitive abilities in low-educated populations. PLoS ONE 2013; 8(3):e60084 10.1371/journal.pone.0060084 23555894 PMC3605367

[28] Choi S, Shim Y, Ryu S, Ryu H, Lee D, Lee J, et al Validation of the Literacy Independent Cognitive Assessment. International Psychogeriatrics 2011; 23 (4):593–601. 10.1017/S1041610210001626 20843392

[29] Pendlebury S, Welch S, Cuthbertson F, Mariz J, Mehta Z, Rothwell P. Telephone assessment of cognition after transient ischemic attack and stroke: modified telephone interview of cognitive status and telephone Montreal Cognitive Assessment versus face-to-face Montreal Cognitive Assessment and neuropsychological battery. Stroke 2013; 44(1):227–9. 10.1161/STROKEAHA.112.673384 23138443 PMC5593099

[30] Mejía S, Miguel A, Villa A, Ruiz L, Gutiérrez L. Deterioro cognoscitivo y factores asociados en adultos mayores en México. Salud Pública Méx 2007; 49(sup 4):475–481. 10.1590/s0036-36342007001000006 17724520

[31] Goh D, Dong Y, Lee W, Koay W, Tay S, Soons D, et al A Pilot study to examine the correlation between cognition and blood biomarkers in a Singapore Chinese male cohort with Type 2 Diabetes Mellitus. PLoS ONE 2014; 9(5): e96874 10.1371/journal.pone.0096874 24816647 PMC4016130

[32] Kodl C, Seaquist E. Cognitive dysfunction and Diabetes Mellitus. Endocr Rev 2008; 29(4):494–511. 10.1210/er.2007-0034 18436709 PMC2528851

[33] Kozora E, Laundenslager M, Lemieux A, West S. Inflammatory and hormonal measures predict neuropsychological functioning in systemic lupus erythematosus and rheumatoid arthritis patients. J Int Neuropsychol Soc 2001; 7:745–754. 11575596 10.1017/s1355617701766106

[34] Moss M, Franks M, Briggs P, Kennedy D, Scholey A. Compromised arterial oxygen saturation in elderly asthma sufferers results in selective cognitive impaired. J Clin Exp Neuropsychol 2005; 27:139–150. 15903147 10.1080/13803390490515450

[35] Marioni R, Stewart M, Murray G, Deary I, Fowkes G, Lowe G, et al Peripheral levels of fibrinogen, C-reactive protein, and plasma viscosity predict future cognitive decline in individuals without dementia. Psychosom Med 2009; 71:901–906. 10.1097/PSY.0b013e3181b1e538 19661193 PMC2762963

[36] Kwasny-Krochin B, Gluszko P, Undas A. Unfavorably altered fibrin clot properties in patients with active rheumatoid arthritis. Thromb Res 2010; 126: 11–16. 10.1016/j.thromres.2010.04.007 20471669

[37] Peters M, Van Halm V, Voskuyl A, Smulders Y, Boers M, Lems W, et al Does rheumatoid arthritis equal diabetes mellitus as an independent risk factor for cardiovascular disease? A prospective study. Arthritis Rheum 2009; 61:1571–1579. 10.1002/art.24836 19877093

[38] Chen Y, Chen H, Lan J, Chen D. Improvement of cognition, a potential benefit of anti-TNF therapy in elderly patients with rheumatoid arthritis. Joint Bone Spine 2010; 77:366–367. 10.1016/j.jbspin.2010.01.017 20478733

[39] Symmons D, Jones M, Scott D, Prior P. Long term mortality outcome in patients with rheumatoid arthritis: Early presenters continue to do well. J Rheumatol 1998; 25:1072–1077. 9632066

[40] Jacobson L, Knowler W, Pillemer S, Hanson R, Pettit D, Nelson R, et al Rheumatoid arthritis and mortality: A longitudinal study in Pima Indians. Arthritis and Rheumatism 1993; 36:1045–1053. 8343181 10.1002/art.1780360804

[41] Walberg S, Öhman M, Dalhqvist S. Cardiovascular morbidity and mortality in patients with seropositive rheumatoid arthritis in northern. Sweden J Rheumatol 1997; 24:445–451. 9058647

[42] Hunt B. The endothelium in atherogenesis. Lupus 2000; 9:189–193. 10805486 10.1191/096120300678828244

